# Characterization of Danube Swabian population samples on a high-resolution genome-wide basis

**DOI:** 10.1186/s12864-022-09092-5

**Published:** 2023-01-09

**Authors:** Zsolt Bánfai, Erzsébet Kövesdi, Katalin Sümegi, Gergely Büki, András Szabó, Lili Magyari, Valerián Ádám, Ferenc Pálos, Attila Miseta, Miklós Kásler, Béla Melegh

**Affiliations:** 1 Department of Medical Genetics, Medical School, University of Pécs, Szigeti út 12, H-7624 Pécs, Hungary; 2 Szentágothai Research Centre, University of Pécs, Ifjúság út 20, H-7624 Pécs, Hungary; 3 Institute of Physiology, Medical School, Hungary, University of Pécs, Ifjúság út 12, H-7624 Pécs, Hungary; 4 Department of Biochemistry and Medical Chemistry, Medical School, University of Pécs, Szigeti út 12, H-7624 Pécs, Hungary; 5 Department of Laboratory Medicine, Medical School, University of Pécs, Ifjúság út 13, H-7624 Pécs, Hungary; 6 National Institute of Oncology, Ráth György u. 7-9, H-1122 Budapest, Hungary

**Keywords:** Genome-wide data, Population genetics, Swabians, Ethnic group, Admixture, Haplotype

## Abstract

**Background:**

German-derived ethnicities are one of the largest ethnic groups in Hungary, dating back to the formation of the Kingdom of Hungary, which took place at the beginning of the 11th century. Germans arrived in Hungary in many waves. The most significant immigration wave took place following the collapse of the Ottoman Empire in East-Central Europe which closed the 150 year long Ottoman occupation. To date, there are no comprehensive genome-wide studies investigating the genetic makeup of the Danube Swabians. Here we analyzed 47 Danube Swabian samples collected from elderly Swabian individuals living in the Dunaszekcső-Bár area, in Danube side villages of Southwest Hungary. These Swabians, according to self-declaration, did not admix with other ethnic groups for 3–6 succeeding generations. Using Illumina Infinium 720 K Beadchip genotype data, we applied allele frequency-based and haplotype-based genome-wide marker data analyses to investigate the ancestry and genetic composition of the collected Danube Swabian samples.

**Results:**

Haplotype-based analyses like identity by descent segment analysis show that the investigated Danube Swabians possess significant German and other West European ancestry, but their Hungarian ancestry is also prominent. Our results suggest that their main source of ancestry can be traced back to Western Europe, presumably to the region of Germany.

**Conclusion:**

This is the first analysis of Danube Swabian population samples based on genome-wide autosomal data. Our results establish the basis for conducting further comprehensive research on Danube Swabians and on other German ethnicities of the Carpathian basin, which can help reconstruct their origin, and identify their major archaic genomic patterns.

**Supplementary Information:**

The online version contains supplementary material available at 10.1186/s12864-022-09092-5.

## Background

The term “Danube Swabians” (also known as “Donauschwaben”) is a collective term for the German-speaking ethnicity populating various countries of East-Central Europe, especially in the valley of the Danube River. While there were German immigrations with smaller numbers also in the 12th century, most of them are the descendants of late 18th century German settlers recruited by the Austrian Empire to repopulate the area and restore agriculture after the defeat of the Ottoman Empire. Danube Swabians can be found in the territory of Hungary, Romania, Serbia, and Croatia.

Hungary has been a multicultural, multiethnic region since the foundation of the former Kingdom of Hungary at the beginning of the 10th century. Germans form the third largest ethnicity in today’s Hungary following the Hungarians and the Romani people. Their estimated number in 2016 was about 178 837 people, which is 1.8% of the total population of Hungary [1].

Germans migrated into the Carpathian basin in multiple waves from several areas [2]. The most significant wave of German immigration, called “the Great Swabian Migration” by historians, originated mostly from the areas of Alsace-Lorraine, Baden, Luxembourg, Pfalz (Mainz), Saarland, Hesse (Frankfurt am Main, Fulda) and Württemberg. This major migration was part of the previously mentioned repopulation project of the Austrians. The German colonization ended in the first half of the 19th century. The population of Swabian settlements remained isolated until their expulsion in 1946, after World War II. The villages, which were almost exclusively German-inhabited villages earlier, became multiethnic. Most of the Danube Swabians assimilated and merged into the Hungarian society during the 19th-20th century.

The village of Dunaszekcső was first settled by Germans in the 18th century with the inhabitants originating from Tyrol, Silesia, Bamberg, Donau-Eschingen, Black Forest, Bavaria and Switzerland, Vienna and Saxony. These settlers dealt mainly with agriculture, handicrafts and viticulture [3]. The population of the village in 2018 was 1892 people with an ethnic composition of Hungarian (80%), German (14%) and Roma (4%) [4]. The first written records mention the neighboring Swabian village Bár (Boor, Bor) in 1296. It was settled by Germans in the 1700s, who established significant vineyards. Most of the settlers came from Southern and Western Germany (Hesse and the area of today’s Baden-Württemberg) [3]. The population of the village was 572 people in 2021, with Hungarian, German, and Serbian ethnicities [4].

The aim of our study was to conduct a pilot genome-wide autosomal investigation of Swabian individuals living in the Dunaszekcső and Bár area (Supplemental Fig. 1), who, according to self-declaration and their reconstructed family trees, are not admixed with members of other ethnicities for 3–6 succeeding generations on their ancestries.

The analysis of isolated populations based on genome-wide autosomal single nucleotide polymorphism data and haplotype data are well-established by several previous studies dealing with isolated Italian populations and populations from the Caucasus region [5–7]. These were comprehensive works, and made also genetic comparison between isolated groups, or even found within-group SNP- and haplotype variations [8]. Due to the relatively small number of available samples at the time, our study concentrates on the obtained Swabian samples as a single homogeneous group, but we intend to compare them also to other isolated and open groups of the European region. In our study, allele frequency and haplotype-based population structure, ancestry estimation software along with formal tests of admixture and identity by descent segment analysis methods were carried out on the genome-wide autosomal marker data of the obtained Danube Swabian samples in order to assess their isolated state and the resulting genetic composition.

## Results

### Population structure and ancestry analysis

**Fig. 1:**
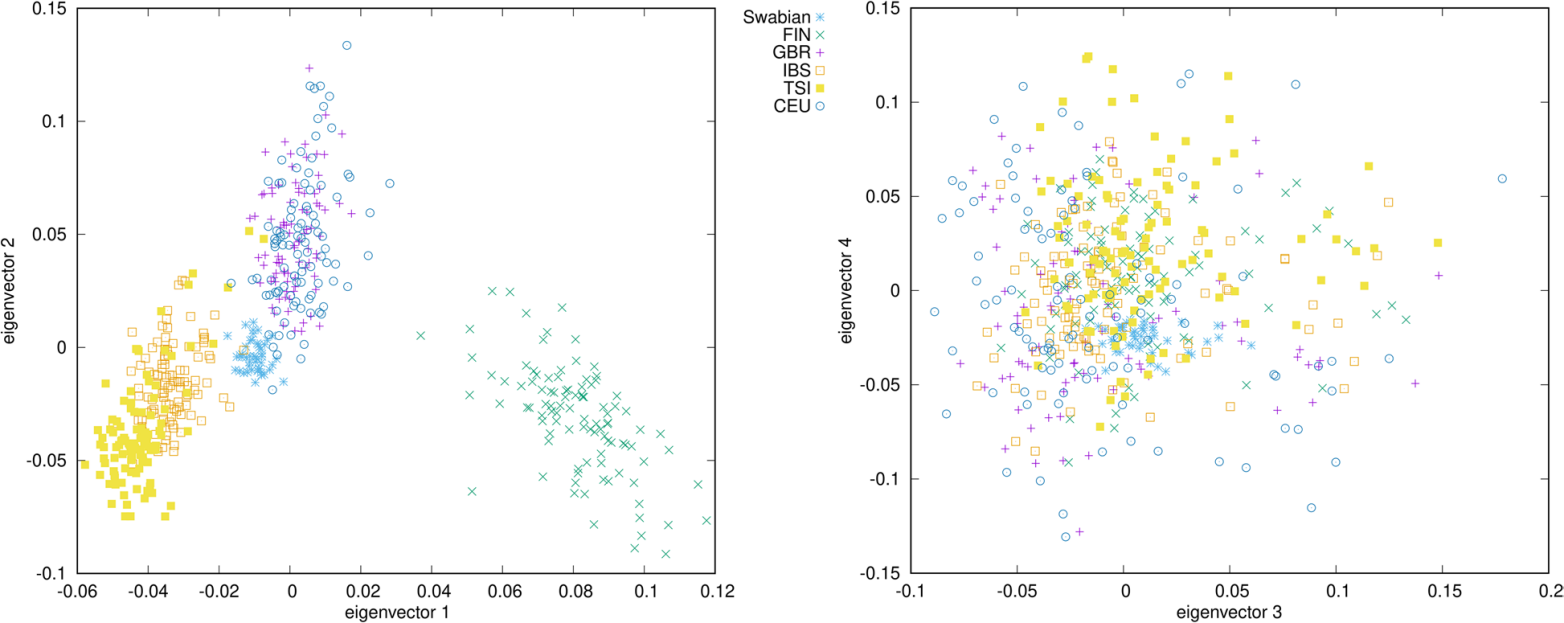
Principal Component Analysis results of Swabians and 1000 Genomes Project populations plotted to the first four principal components. Eigenvalues of eigenvectors 1, 2, 3 and 4 were 2.647, 1.590, 1.419 and 1.388, respectively. 353 of the calculated eigenvectors had eigenvalues higher than 1.000. Each symbol represents one individual. GBR – British from England and Scotland; FIN – Finnish in Finland; IBS – Iberian populations in Spain, TSI – Toscani in Italy; CEU – Utah residents with Northern and Western European ancestry from the CEPH collection

**Fig. 2:**
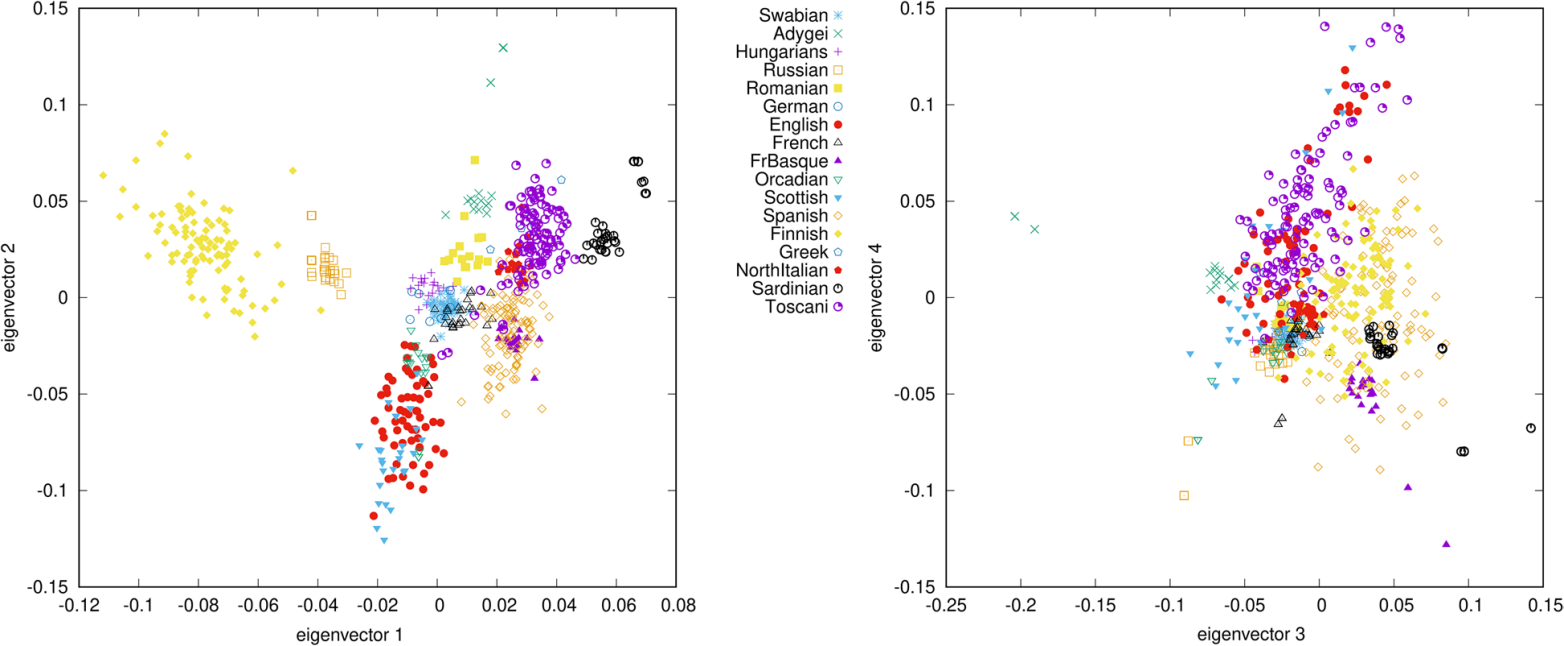
Principal Component Analysis results of Swabians and various European populations plotted to the first four principal components. Eigenvalues of eigenvectors 1, 2, 3 and 4 were 3.986, 2.420, 2.040 and 1.688, respectively. 531 of the calculated eigenvectors had eigenvalues higher than 1.000. Each symbol represents one individual

Principal component analysis of Swabians and European 1000 Genomes Project (1KGP) [9] populations show that Swabians belong to the West European cluster along with British and CEU samples but shows also other European genetic components which is indicated here by the orientation of their clustering towards South Europe (Toscani and Spanish) (Fig. 1). PCA results investigating various European populations show that Swabians samples are rather tightly clustered with each other (Fig. 2). Europeans consist of some subgroups, which are East and North Europeans and West and South Europeans. Sardinians form a completely separate group, which might indicate their genetic isolation [10]. In the European cluster, Swabians are plotting together with West Europeans, like French and Germans and they are also considerably close to Hungarians. South, East and North Europeans form rather a different subgroup. The French Basques also constitute a separate subgroup clustering weakly together with the Spanish samples, which also indicates isolation, similar to the Sardinians.

**Fig. 3:**
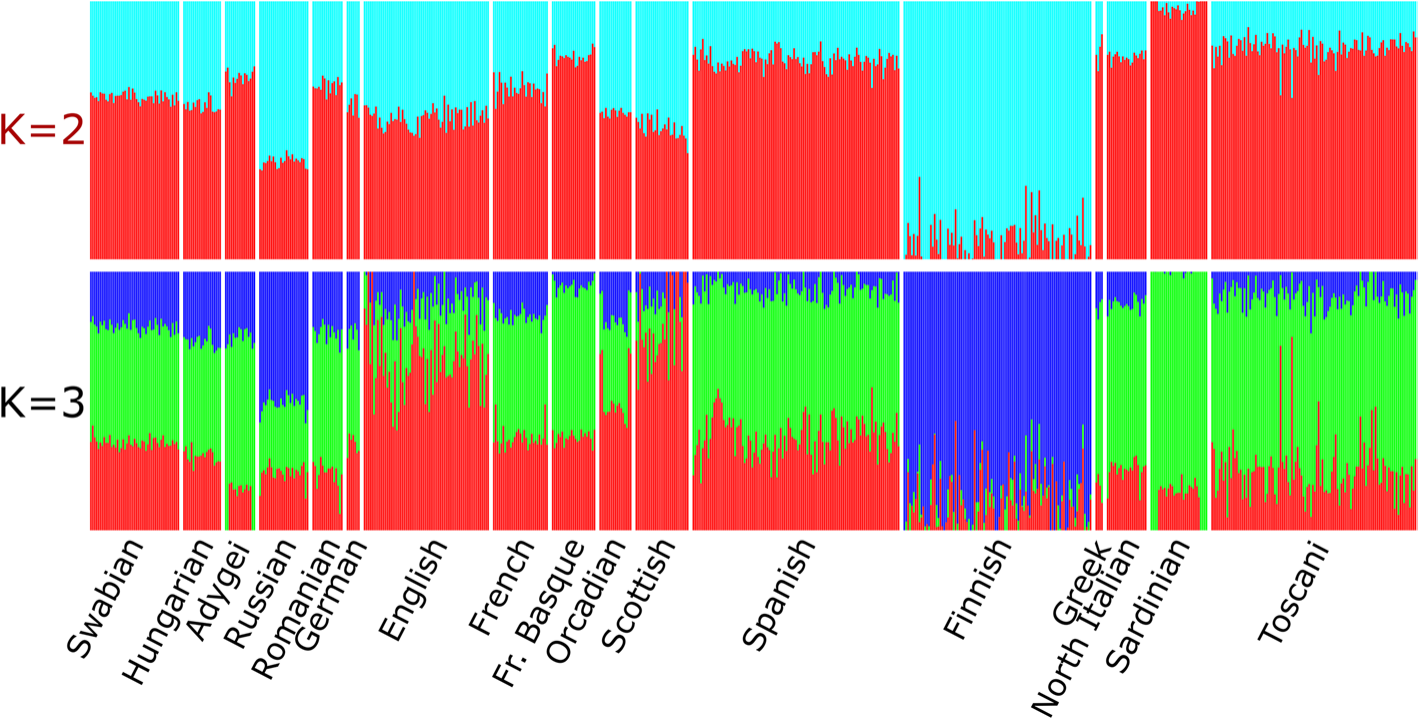
ADMIXTURE analysis results of Swabians and investigated European populations with K = 2–3. Cross-validation error was the lowest applying 2 clusters. Cross-validation error values at K = 2, K = 3 was 0.86212 and 0.86379, respectively. The cross-validation error data can be found on Supplemental Fig. 2. Each column represents one individual, each column group represents a population

In the ADMIXTURE analysis of Swabians and various European populations, the cross-validation error was the lowest at K = 2. The resulting ADMIXTURE graph strengthens the results of PCA and reflects a very similar relationship of the investigated populations (Fig. 3). According to ADMIXTURE, ancestry proportions of Swabians from the two clusters are most similar to the West European populations, especially the French, the Germans and the Orcadians and show also high similarity to Hungarians. The phenomenon can be observed on the ADMIXTURE graph featuring 3 clusters. ADMIXTURE analysis results with K = 2 to K = 8 clusters and the calculated cross-validation error values can be found in the supplemental data (Supplemental Figs. 2 and 3).

**Fig. 4:**
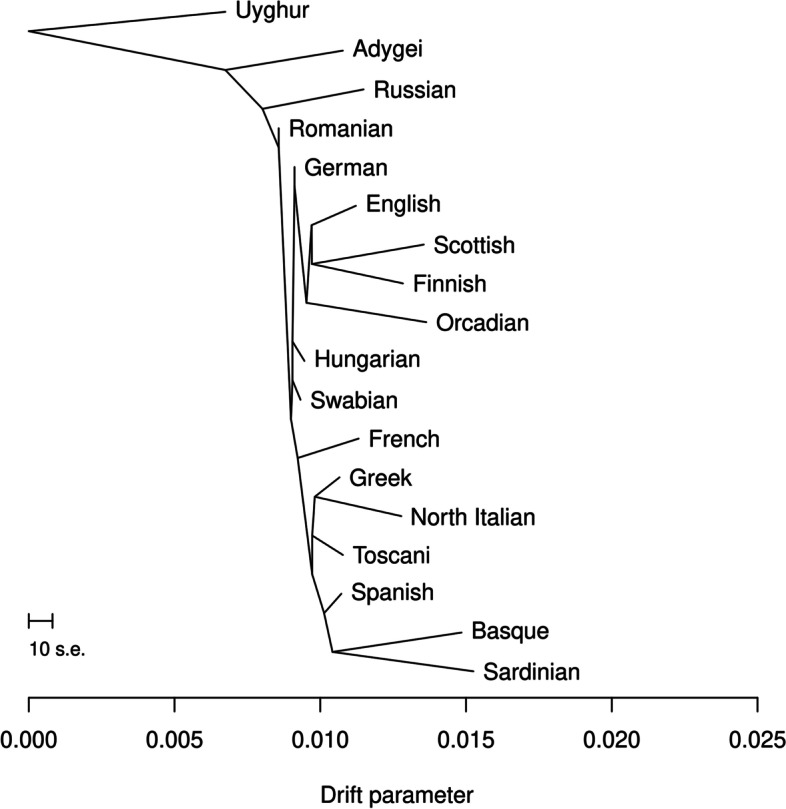
TreeMix analysis results. The calculated maximum likelihood tree. Residual fit of this analysis showing the standard error of the calculations can be found on Supplemental Fig. 4

TreeMix also reflects the same relationships, as the drift parameter is most similar in case of West Europeans to Swabians within the branch consisted of Europeans (Fig. 4, Supplemental Fig. 4). TreeMix was not able to detect any migration events between the investigated populations.

**Fig. 5:**
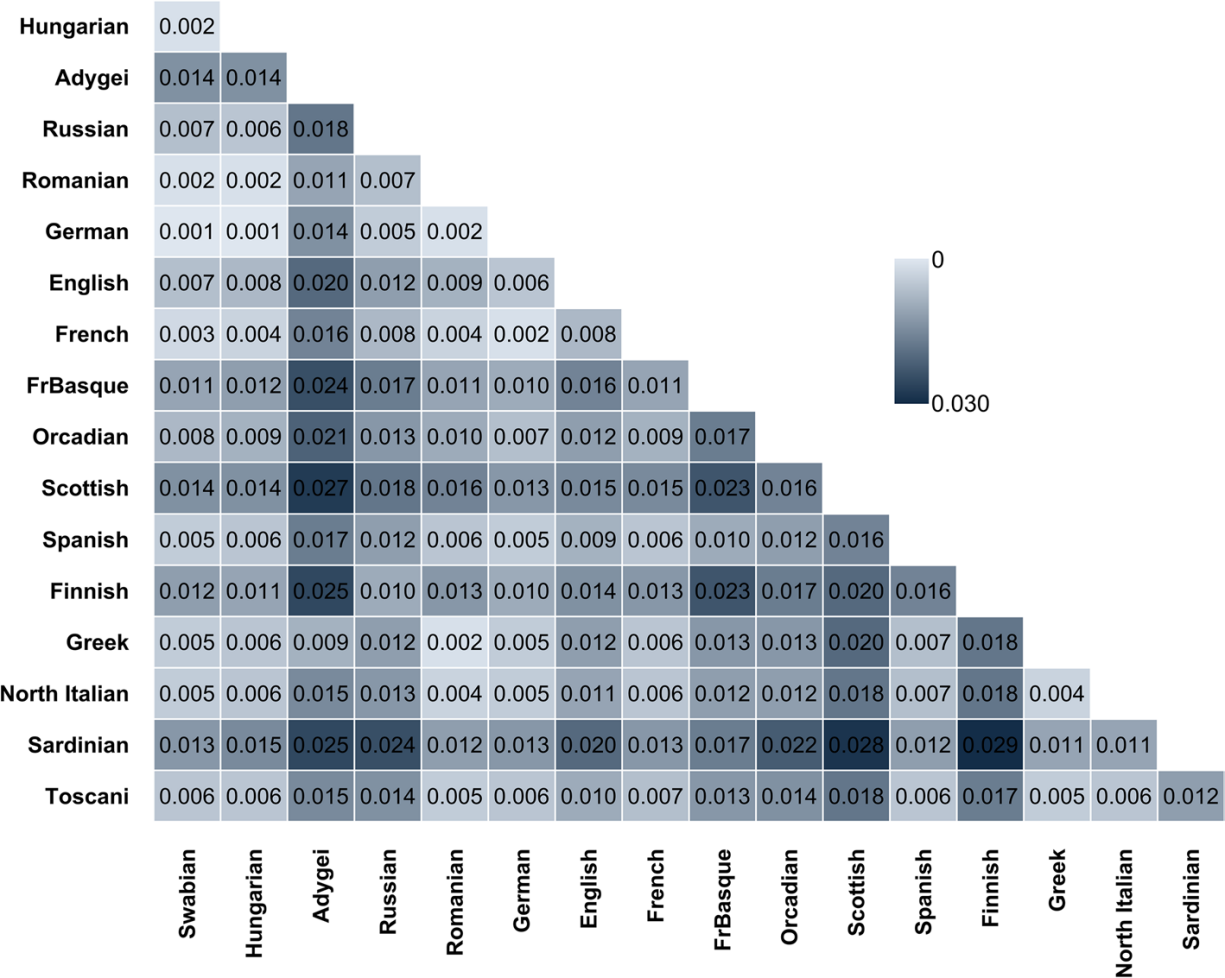
F_st_ (Fixation index) matrix calculated by the SMARTPCA software, showing the average pairwise allele frequency differentiations between the investigated Danube Swabians and various European populations. Standard error of the F_st_ calculations can be observed at the Supplemental Fig. 5

The average pairwise allele frequency differentiation matrix (F_st_ matrix) quantifies the results of PCA and shows that Swabians are similarly close to Hungarians, Germans and French, but they have the lowest fixation index value with the German samples (Fig. 5, Supplemental Fig. 5).

### Formal test for assessing admixture

The 4-population test show that genetic relationship of Swabians and Germans are stronger than the relationship of almost any European groups except of Hungarians. (Table 1). However, D-statistics do not give significant Z-scores in case of Germans and Hungarians indicating that genetic relationship of Swabians to Hungarians and to Germans might be of a similar magnitude.

**Table 1:** D-statistics results

Outgroup	Population 1	Population 2	Population 3	D-statistics	Z-score
W	X	Y	Z		
YRI	Hungarian	Swabian	German	0.0000	-0.0130
YRI	Swabian	Hungarian	German	0.0002	0.1800
YRI	German	Swabian	Hungarian	-0.0002	-0.2500
YRI	English	Swabian	Hungarian	-0.0014	-2.0620
YRI	French	Swabian	Hungarian	-0.0010	-1.5780
YRI	Orcadian	Swabian	Hungarian	-0.0013	-1.8780
YRI	Scottish	Swabian	Hungarian	-0.0011	-1.6860
YRI	Spanish	Swabian	Hungarian	-0.0014	-2.2200
YRI	N_Italian	Swabian	Hungarian	-0.0010	-1.5530
YRI	Toscani	Swabian	Hungarian	-0.0008	-1.3110
YRI	Russian	Swabian	Hungarian	0.0010	1.5100
YRI	Romanian	Swabian	Hungarian	0.0000	-0.0500
YRI	English	Swabian	German	-0.0008	-0.9840
YRI	French	Swabian	German	-0.0011	-1.3480
YRI	Orcadian	Swabian	German	-0.0007	-0.8380
YRI	Scottish	Swabian	German	-0.0006	-0.7280
YRI	Spanish	Swabian	German	-0.0017	-2.1970
YRI	N_Italian	Swabian	German	-0.0018	-2.1820
YRI	Toscani	Swabian	German	-0.0020	-2.5480
YRI	Russian	Swabian	German	0.0013	1.5870
YRI	Romanian	Swabian	German	-0.0007	-0.9000

### Detailing relationships using haplotype-based analyses

**Fig. 6:**
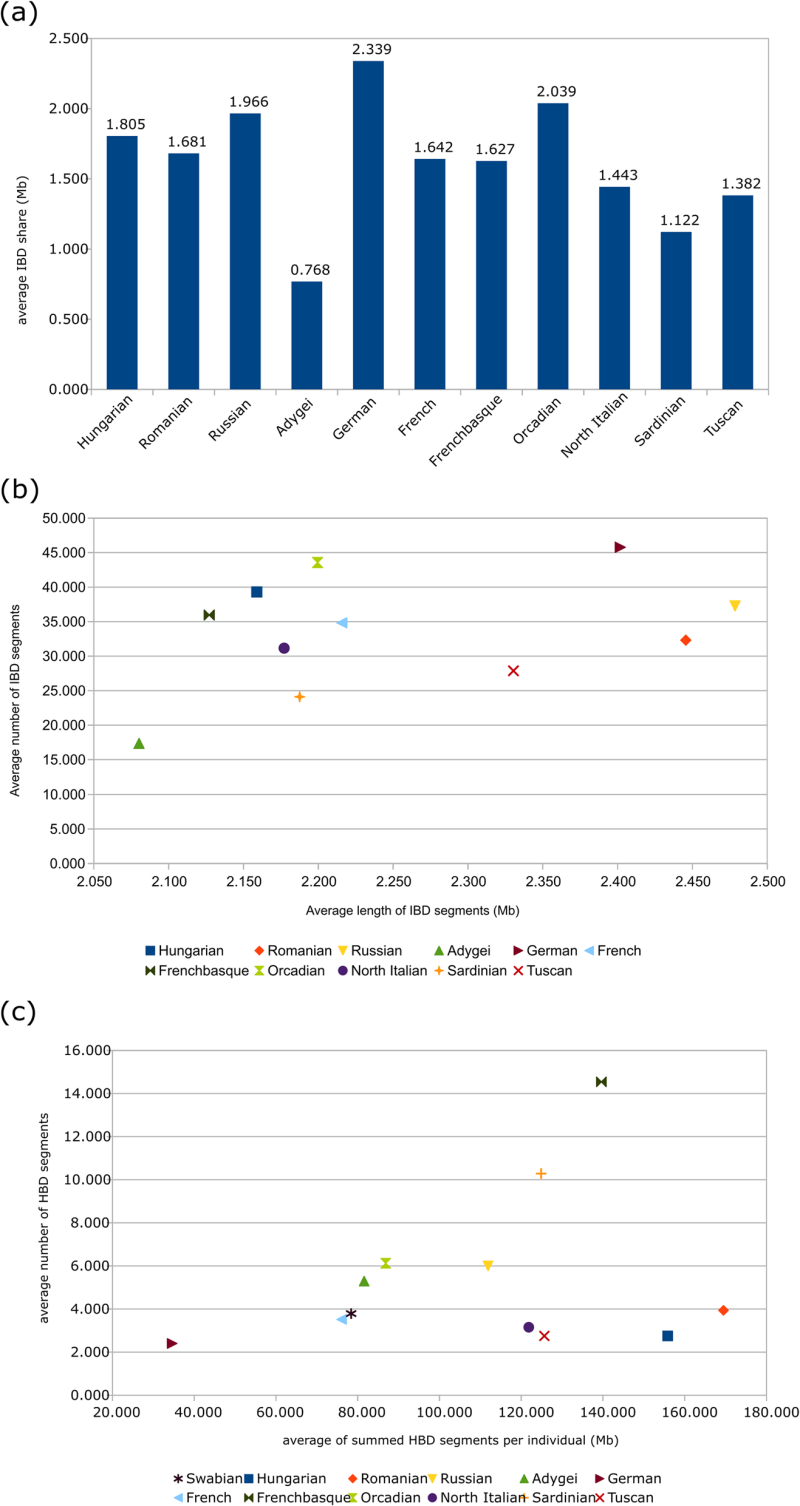
Results of haplotype-based investigations. **a** Average pairwise IBD sharing results, **b** Average length and number of shared IBD segments between Danube Swabians and other investigated groups, **c** Average genome-wide autozygozity calculations of investigated populations

The average identity by descent (IBD) share of Swabians is the lowest with Adygei (0.768) and Sardinians (1.122) (Fig. 6a). Average IBD share of Swabians is the highest with Germans, which is 2.339 and with Orcadians with an average share of 2.039. The average IBD share of Swabians with Hungarians is 1.966. This analysis points out that Germans are the most important ancestry source of Swabians, but results concerning Hungarians and other West European populations, namely the Orcadians show, that they are also an important source of ancestry of the investigated Danube Swabians.

Average length and number of IBD segments show that Hungarians are very similar to Germans in this regard, but average length of shared IBD segments of Swabians with Germans are relatively large, which agrees with the average IBD sharing results (Fig. 6b). The average genome-wide autozygosity is similar to most investigated populations, and much smaller than known isolated groups like Sardinian and French Basques (Fig. 6c). This relatively low number and relative shortness of HBD segments show that inbreeding is not observable in the Swabian samples.

## Discussion

Our population structure analysis with PCA points out that our Danube Swabian samples are the closest to West Europeans in the European cluster according to the analysis using the 1KGP data. Applying various European populations, these analyses show that Swabians are similarly close to Hungarians and Germans. This suggests that the ancestry of Swabians includes Germans and Hungarians as well at similar proportions. In agreement with the 1KGP data analysis, Swabians are closest to the Western European populations as we can see in the case of Orcadian and French samples. Using three clusters in the ADMIXTURE analysis, investigated populations share three sources of common ancestry, of which two are major clusters with the highest ancestry proportions. According to ADMIXTURE, the share from these ancestries in case of Swabians, Hungarians and Germans are quite similar, which was shown also by the similar F_st_ values. However, F_st_ calculations indicate the lowest average pairwise allele frequency differentiation between the Swabians and the Germans. With a third ancestry estimation method, which also utilizes a maximum likelihood method as the algorithm of ADMIXTURE, the genetic differences between Hungarians and Swabians became more pronounced and mirrored the results of PCA, Swabians show to be closer to West Europeans rather than Hungarians.

Using formal tests of admixture, we further investigated the relationship of Swabians, Germans and Hungarians. These tests were unable to highlight a more significant relationship between Germans and Hungarians as the tests showed no significance. However, these tests strengthened the results that our Danube Swabian samples are the closest to the Germans besides Hungarians among the populations used in this study.

Investigating the source of ancestry of the Swabian group, calculating the average pairwise IBD share between Swabian individuals and samples from the other investigated populations show that the most important source of ancestry indeed comes from Germany. However, Hungarians show also a relatively high amount of IBD share with Swabians. Average IBD sharing results point out that the main source of the Swabian ancestry is West Europe followed by Eastern European samples. South Europeans show to be the least important source of Swabian ancestry. Studying the average number and average length of shared IBD segments between Swabians and investigated populations, both the average number and average length of shared IBD segments are higher in the case of the Germans in contrast of Hungarians. Although, our Danube Swabian samples might belong to a socially closed population, average genome-wide autozygosity calculations do not indicate isolation in Swabians. This was strengthened by the fact that known isolated ethnic groups like the French Basques and also the Sardinians possess much higher autozygosity than of Swabians, who are hardly standing out from the average in this regard. This high genetic isolation of the French Basques, observed also in our tests, was caused by Post-Iron Age demographic isolation processes [11, 12]. Moreover, HBD segment analysis points out that Swabians do not show the signs of inbreeding either.

Our future goal in this topic is to obtain a far larger set of samples including Danube Swabians, other Swabians and other German-derived ethnicities and besides managing them as a homogeneous group, which is a usual method in the study of isolated populations, we plan to investigate also their within-population individual diversity to study possible intra-population structure and to carry out a large scale comparative study of these populations seen in the paper Anagnostou et al. 2019 [13].

## Conclusion

According to the average genome-wide autozygosity of Swabians, they do not show isolation compared to other ethnic groups, especially comparing with known isolated populations. They show to be closer to Germans and West Europeans, but their relationship with Hungarians is also strong.

The Danube Swabian population of Dunaszekcső and Bár show indeed strong connections with Germans but are also well-relatable to Hungarians, which suggest admixture with them at some point in history. Our D-statistics result and IBD analysis results might suggest this admixture event, but in order to support it with a plausible model, we need to find appropriate surrogate populations for the contemporary groups involved in this admixture event.

With the help of genome-wide autosomal marker data, we were able to assess the significance of German and West European derived ancestry in the Danube Swabians samples and pointed out that Hungarians also play an important role in their ancestry. These carefully selected samples are from Swabian individuals born in the first half of the 20th century when ethnic self-awareness and the preservation of their specific culture and heritage were part of their everyday life. In the late 20th and in the 21st centuries these traditions became less and less commonplace shrinking back to special cultural events rather than being an everyday lifestyle, and the Swabian population began also to mingle more significantly with other surrounding ethnicities. Their lives also shifted towards cities which further facilitated the continuous decline of the population of traditional Swabian villages. These processes render our Swabian sample collection invaluable and should be the basis of further, much more detailed investigations regarding the German derived ethnicities of the Carpathian basin.

## Methods

### Samples and applied data

In this study we examined samples from 47 Danube Swabian individuals with well-documented family history dating back to 3–6 succeeding generations with unadmixed Swabian ancestries supported by self-declaration-based family history and the resulting pedigree trees. The sampled Danube Swabian individuals live in the villages of Dunaszekcső and Bár which can be found along the Danube River in Southwest Hungary (Supplemental Fig. 1). 29 samples are from Dunaszekcső and 18 samples were collected from the village of Bár. From the 47 individuals, 19 were males and 28 were females, so the M/F ratio was 0.68. The Swabian population of these villages remained mostly isolated from other ethnicities until today, providing an opportunity to study their genetic makeup and relationship with major European groups.

DNA was extracted from ethylenediaminetetraacetic acid (EDTA)-anticoagulated whole blood and was genotyped on the Illumina Infinium Global Screening Array Beadchip platform which contains 725 831 single-nucleotide polymorphisms (SNPs). Isolation, genotyping, and preliminary quality control of the samples was carried out by the third-party service provider Human Genomics Facility (HUGE-F) in the Netherlands at the University of Rotterdam. Quality control and data preparation of the marker data was carried out domestically applying in-house scripts and the PLINK1.9 and 2.0 software packages [14, 15]. The data was filtered using the Hardy-Weinberg equilibrium tests, and additionally, SNPs with missing genotypes were removed from the dataset using PLINK with the ‘geno’ flag applying a threshold value of 0.1. All Swabian individuals passed these tests and 665 073 SNPs remained in our Danube Swabian dataset.

This study belongs to a series of investigations that were approved by the National Ethics Board (ETT TUKEB), and by Regional Ethics Committee of Pécs and follows the principles expressed in the Declaration of Helsinki.

Genome-wide autosomal marker data from other open genotype databases was also considered in the study. We used the 1000 Genomes Project (1KGP) and Human Genome Diversity Project (HGDP) datasets which are openly available from the respected sources [9, 16–18]. We also considered population data from datasets of the open genome-wide marker data repository which can be found on the server of the Estonian Biocentre [19, 20]. We used also the Allen Ancient DNA Resource (AADR) dataset which is openly available from the David Reich lab on the Harvard University [21]. Populations from the European and Caucasus regions were applied from the HGDP and 1KGP datasets. Additional populations from the Estonian Biocentre included Hungarians, Romanians, and Germans. German samples were filtered according to preliminary PCA and ADMIXTURE analyses using 1KGP and HGDP data separately, since we discovered that some of the German samples are outliers possessing significant non-West European (presumably East European) genetic ancestry. These German samples (6) were removed from the German data prior to our analyses. Since the sampling of German data was based on self-declaration, some of these individuals might not originate from the area of Germany but from neighboring countries.

### Principal component analysis-based population structure analysis

Population structure analysis along with fixation index (F_st_) matrix calculation were achieved using the SMARTPCA software of the EIGENSOFT 6.1.4 package [22].

For the PCA analysis, first, we merged the Swabian samples with European 1KGP groups, namely with British (English and Scottish) samples from England and Scotland (GBR), Finnish from Finland (FIN), Iberian samples from Spain (IBS), Toscani from Italy (TSI) and Utah residents with Northern and Western European ancestry from the collection of CEPH (CEU).

A second merged dataset containing the Swabian samples with various European groups using HGDP, AADR and Estonian Biocentre data was also created and analyzed using PCA. This dataset includes the HGDP populations French, French Basques, Orcadians, North Italians, Sardinians, Tuscans, Russians and Adygei. Populations from the AADR were the English, Scottish, Spanish, Finnish, and Greek, including also 1KGP samples of these groups. From the Estonian data, Hungarians, Romanians, and Germans were used.

The first, 1KGP dataset contained *n* = 556 individuals and 159 240 SNPs, the secondly created dataset featuring various European populations from various repositories contained *n* = 666 individuals and 106 121 SNPs. SNPs with strong background linkage disequilibrium (LD) were also pruned out with the ‘indep-pairwise’ command of PLINK1.9 setting the *r*^2^ threshold to 0.3. It is necessary before the analyses due to strong background LD can bias the PCA method, but also expectation maximization-based ancestry estimation algorithms like ADMIXTURE and TreeMix which were used in this study. After the pruning process, 149 979 and 79 757 SNPs remained in our first and second dataset, respectively. We used SMARTPCA with default settings, the σ-threshold was set to 6.0. F_st_ calculations were carried out with our second dataset using the “fstonly” option of the SMARTPCA software.

### Maximum likelihood method-based ancestry estimation

Ancestry estimation was carried out with the ADMIXTURE 1.22 algorithm which is a maximum likelihood estimation method using an expectation maximization approach [23]. We carried out ADMIXTURE analysis on our second dataset containing various European populations from different genotype data repositories. The correct number of clusters (K) were calculated applying K values of 2 to 10 and cross-validation was also performed in order to find the best fitting K for the relationship of our investigated populations.

TreeMix was also applied along with ADMIXTURE analysis on this dataset, to better describe the relationship of these populations in a maximum-likelihood tree-based manner in addition to the stacked column styled ancestry estimation [24]. The size of the SNP blocks (-k flag) was set to 1000 and we also set the algorithm to estimate for 1–6 migration events in the data through multiple runs. For these investigations, the same pruned dataset was used that was created for PCA, but Uyghurs from the HGDP data was added as outgroup (*n* = 681, 79 757 SNPs).

### Formal test of admixture

In order to test the relationship of Swabians and other investigated populations in the second dataset, we utilized a formal test of admixture, the 4-population test. The qpDstat program from the ADMIXTOOLS 4.1 package was used for this purpose, and as its name suggests, this test was implemented here as D-statistics [25]. For these calculations, we used the unpruned version of our second dataset. YRI from the 1KGP data was added to these tests as an outgroup. We tested the unrooted phylogenetic trees containing YRI, Swabians, Hungarians and various European populations, Germans, English, French, Orcadian, Scottish, Spanish, North Italian, Toscani, Russian and Romanian. We applied five different setups of the ((W,X)(Y,Z)) unrooted trees which were the following:

((YRI,Hungarian)(Swabian,German)), ((YRI,Swabian)(Hungarian,German)), ((YRI,European Test)(Swabian,Hungarian)), ((YRI,European Test)(Swabian,German)). These tests intended to show the relationship of Swabians to the Hungarian host population, to the Germans and to various European populations.

### Identity by descent and homozygous by descent analyses

For assessing the sources of ancestry in the investigated Swabian samples, we implemented here the Refined IBD algorithm of Beagle 4.1 [26]. The software seeks in phased haplotype data for IBD segments between all pairs of individuals, which shows us the relative share of one population in the ancestry of the investigated population. In order to minimalize the SNP loss, we used in this test an unpruned dataset consisting only of Swabians and the HGDP and Estonian Biocentre groups, featuring *n* = 601 individuals and 110 733 SNPs. Before the analysis, the data was converted according to the needs of the algorithm using the PLINK1.9 software. The major alleles were set as A2 allele and the dataset was converted to Variant Call Format 4.1 with the PLINK/SEQ software [27]. The minimum segment length was set to 3 centiMorgan, the IBD trim parameter value was 10. The IBD scale parameter was calculated with the $\sqrt{n/100}$ recommended formula since our data contained more than 400 individuals [26]. Using the inferred IBD segment data, we calculated an average pairwise IBD sharing between Swabians and various populations with the following formula according to Atzmon et al.:

$$Average\;pairwise\;IBD\;sharing=\frac{\sum_{i=1}^n\sum_{j=1}^m{IBD}_{ij}}{n\cdot m}$$

IBD_ij_ is the length of the IBD segment shared between individuals i and j. The n and m are the number of individuals in the groups I and J [28].

We also calculated the average number and average length of IBD segments between Swabians and the investigated various populations.

Besides IBD segments, Refined IBD simultaneously detects homozygous by descent (HBD) segments, which allows us also to infer the genome-wide autozygosity of respective populations. This can imply the degree of isolation and degree of inbreeding of these groups. Therefore, average length and number of HBD segments were also calculated.

## Supplementary Information

**Additional file 1.**

**Additional file 2.**

**Additional file 3.**

**Additional file 4.**

**Additional file 5.**

## Data Availability

All data generated or analyzed during this study are included in this published article and its supplementary figures. All analyzed datasets are available in public online repositories, except the Danube Swabian data which according to the Hungarian Human Genetics Act 2008/XXI, cannot be uploaded to a public online database, but can be obtained upon reasonable request via e-mail from the corresponding authors.

## Abbreviations

AADR: Allen Ancient DNA Resource
1KGP: 1000 Genomes Project
EDTA: Ethylenediaminetetraacetic acid
HBD: Homozygous by descent
HGDP: Human Genome Diversity Project
IBD: Identity by descent
LD: Linkage disequilibrium
PCA: Principal component analysis
SNP: Single-nucleotide polymorphism
