# Supplementary material for: Characterization of Danube Swabian population samples on a high-resolution genome-wide basis
Source: BMC Genomics. 2023 Jan 9;24:9. doi: 10.1186/s12864-022-09092-5 (PMC9830925; doi:10.1186/s12864-022-09092-5)
Supplement: Supplementary file 1 — Additional file 1. [file 12864_2022_9092_MOESM1_ESM.pdf]

Supplemental Figure 1. Area of origin of the Danube Swabian samples. The Dunaszekcső-Bár area.

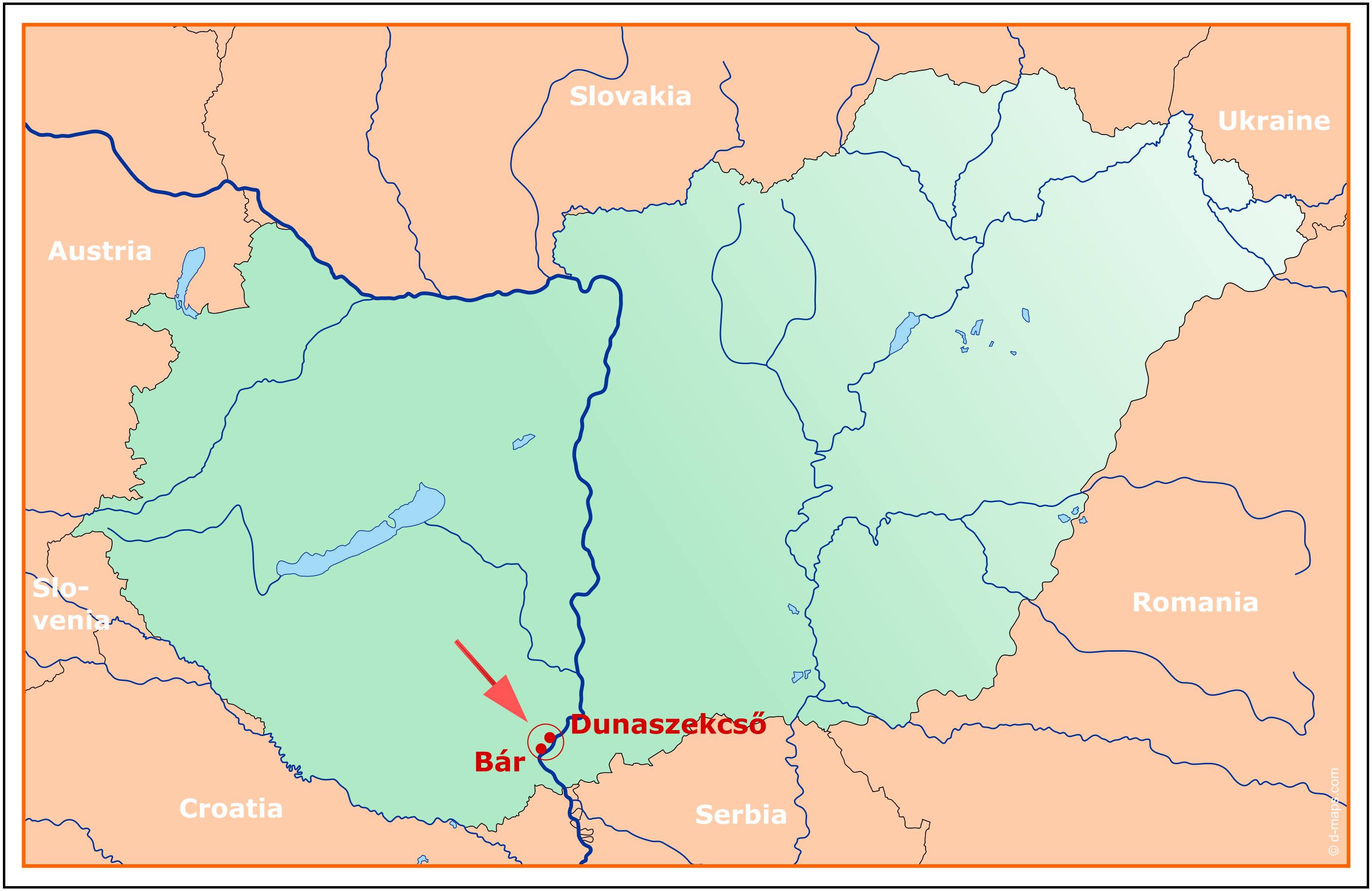

Source of the original image: https://d-maps.com/carte.php?num_car=3562&lang=en
