# Supplementary material for: Characterization of Danube Swabian population samples on a high-resolution genome-wide basis
Source: BMC Genomics. 2023 Jan 9;24:9. doi: 10.1186/s12864-022-09092-5 (PMC9830925; doi:10.1186/s12864-022-09092-5)

Supplemental Figure 2. Cross-validation of the ADMIXTURE analysis runs (dataset: Swabians and Eurasian populations).

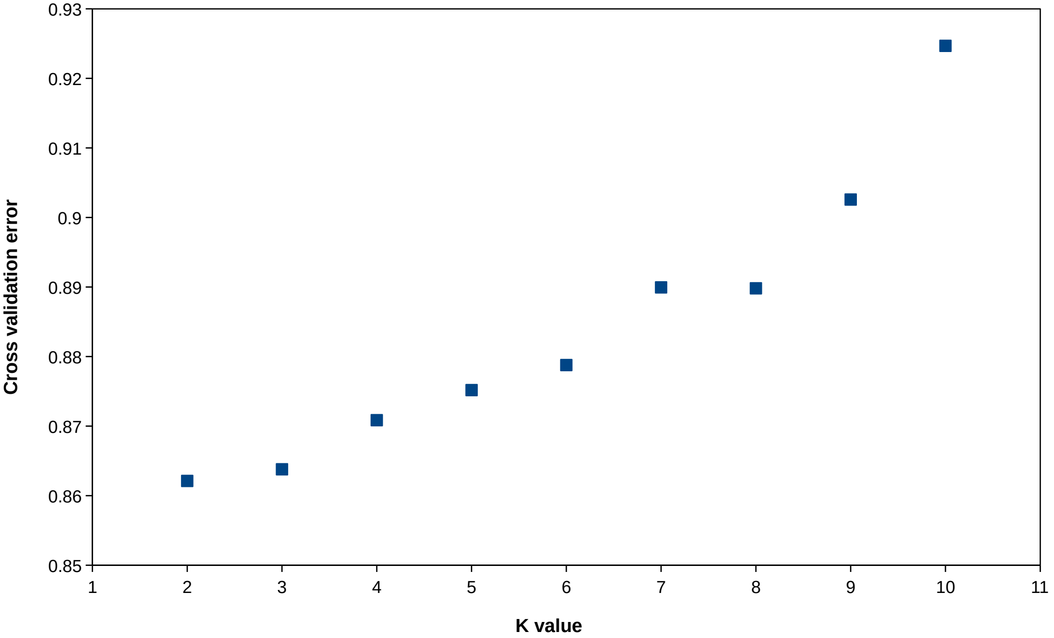

Supplement: Supplementary file 2 — Additional file 2. [file 12864_2022_9092_MOESM2_ESM.pdf]
