# Supplementary figures and images for: Characterization of Danube Swabian population samples on a high-resolution genome-wide basis
Source: BMC Genomics. 2023 Jan 9;24:9. doi: 10.1186/s12864-022-09092-5 (PMC9830925; doi:10.1186/s12864-022-09092-5)

**Supplemental Figure 3.** Admixture analysis of Swabians and other Europeans using 2-8 K-values.

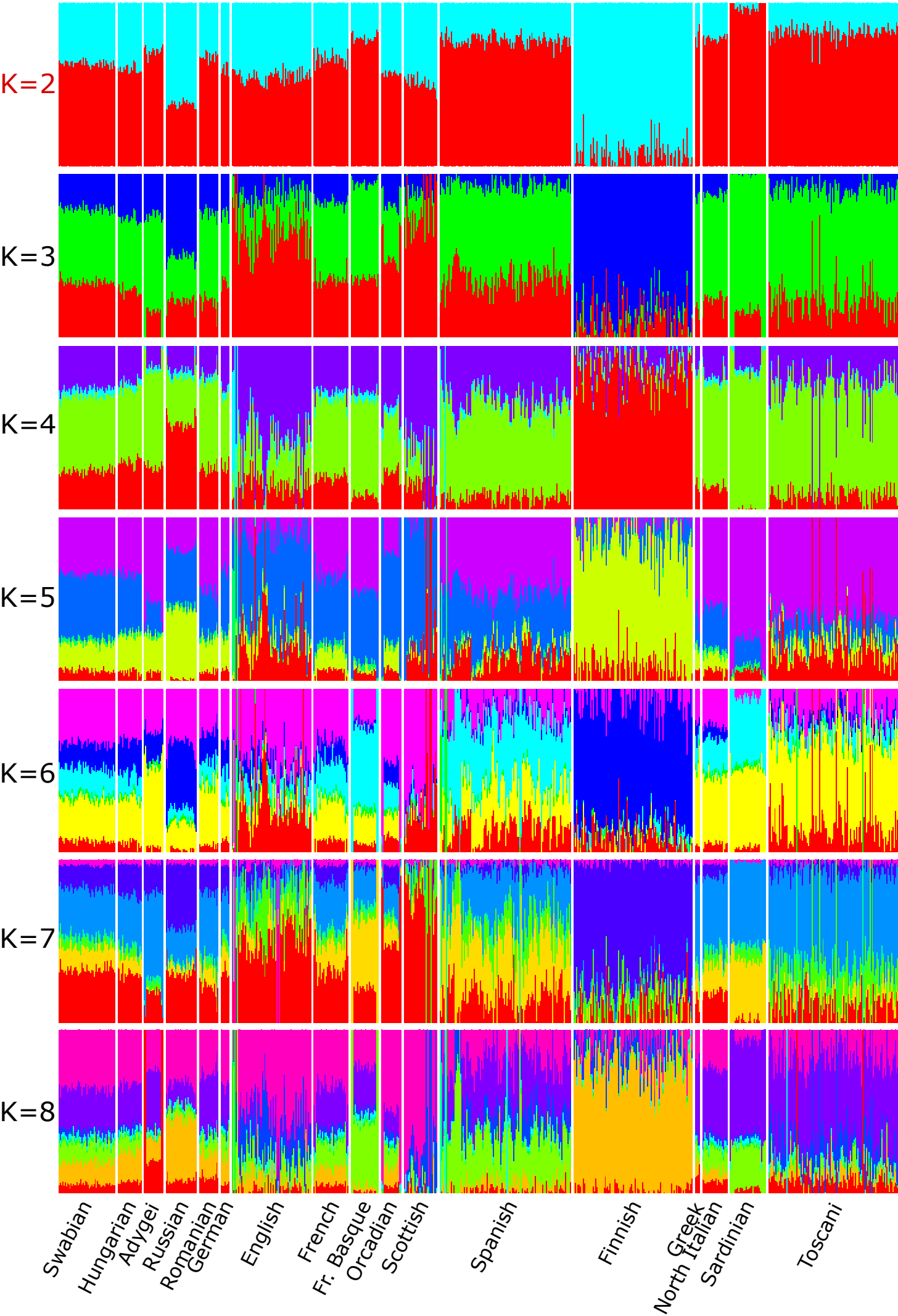

Supplement: Supplementary file 3 — Additional file 3. [file 12864_2022_9092_MOESM3_ESM.pdf]

**Supplemental Figure 4.** Residual Fit of the TreeMix analysis results.

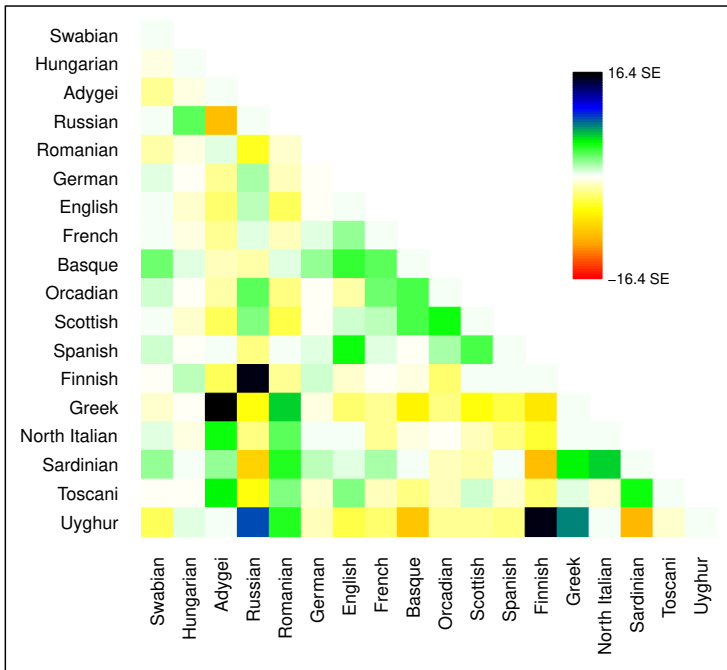

Supplement: Supplementary file 4 — Additional file 4. [file 12864_2022_9092_MOESM4_ESM.pdf]
