# Supplementary material for: Characterization of Danube Swabian population samples on a high-resolution genome-wide basis
Source: BMC Genomics. 2023 Jan 9;24:9. doi: 10.1186/s12864-022-09092-5 (PMC9830925; doi:10.1186/s12864-022-09092-5)
Supplement: Supplementary file 5 — Additional file 5. [file 12864_2022_9092_MOESM5_ESM.pdf]

Supplemental Figure 5. Standard error values of the F_{st} matrix of Swabians and various European populations calculated with the Eigensoft software package.

	Swabian	Hungarian	Adygei	Russian	Romanian	German	English	French	FrBasque	Orcadian	Scottish	Spanish	Finnish	Greek	North Italian	Sardinian
**Hungarian**	154															
**Adygei**	278	345														
**Russian**	170	211	345													
**Romanian**	172	223	292	251												
**German**	308	371	484	386	383											
**English**	103	180	303	180	190	337										
**French**	124	191	300	207	208	355	136									
**FrBasque**	214	311	405	313	284	436	235	241								
**Orcadian**	219	293	403	295	310	447	227	253	337							
**Scottish**	177	235	371	242	263	396	167	202	301	275						
**Spanish**	89	170	267	182	163	319	99	126	182	224	167					
**Finnish**	149	217	347	164	227	336	136	181	301	253	210	158				
**Greek**	578	647	649	640	596	756	570	582	677	682	684	547	615			
**North Italian**	172	243	301	263	228	384	192	208	258	310	265	144	242	575		
**Sardinian**	230	309	366	344	293	446	264	242	294	391	336	189	349	650	256	
**Toscani**	106	163	237	201	150	321	120	127	204	238	195	71	172	531	139	176

10

800

Stdev · 1000000
